# COMMENCE trial (Comparing hypOtherMic teMperaturEs duriNg hemiarCh surgEry): a randomized controlled trial of mild vs moderate hypothermia on patient outcomes in aortic hemiarch surgery with anterograde cerebral perfusion

**DOI:** 10.1186/s13063-019-3713-9

**Published:** 2019-12-09

**Authors:** Habib Jabagi, George Wells, Munir Boodhwani

**Affiliations:** 1 Divisions of Cardiac Surgery, University of Ottawa Heart Institute, 40 Ruskin Street, Room H-34058A, Ottawa, ON K1Y 4W7 Canada; 2 Cardiovascular Research Methods Centre, University of Ottawa Heart Institute, 40 Ruskin Street, Ottawa, ON K1Y 4W7 Canada

**Keywords:** Aortic arch surgery/hemiarch repair, Hypothermic circulatory arrest, Mild hypothermia, Antegrade selective cerebral perfusion, Cerebral protection, Stroke, Acute kidney injury, Prospective study, Randomized controlled trial

## Abstract

**Background:**

Aortic arch surgery remains the only viable life-saving treatment for aortic arch disease. However, the necessity for cessation of systemic blood flow with hypothermic cardiac arrest carries substantial risk of morbidity and mortality, including poor neurological outcomes and kidney failure. While uncontrolled studies have suggested the safety of operating at warmer temperatures, significant variables remain un-investigated, supporting the need for a randomized clinical trial (RCT) to produce evidence-based guidelines for perfusion strategies in aortic surgery. This study proposes a multi-center RCT in order to compare outcomes of warmer hypothermic strategies during aortic hemiarch surgery on a composite endpoint of neurologic and acute kidney injury (AKI).

**Methods/design:**

This is a prospective multi-center, single-blind two-arm RCT comparing mild (32 °C) versus moderate (26 °C) hypothermic cardiac arrest in patients (*n* = 282) undergoing hemiarch surgery with antegrade cerebral perfusion (ACP). The primary endpoint is a composite of neurological injury (incidence of transient ischemic attack and/or stroke) and Kidney Disease Improving Global Outcomes (KDIGO) stage 1 or higher AKI. Secondary outcomes include death, cardiopulmonary bypass time, bleeding, transfusion rates, prolonged mechanical ventilation, myocardial infarction, length of stay, and quality of life measures.

Patients will undergo 1:1 block randomization to each treatment arm on day of surgery. Sequence of operation will be at the surgeon’s discretion with mandatory guidelines for temperature and ACP administration. Perioperative management will occur as per enrolling center standard of care. Neurocognitive function will be assessed for neurological injury using validated neurological screening tests: NIHSS, MOCA, BI, and MRS throughout patient follow-up. Diagnosis and classification of AKI will be based on rising creatinine values as per the KDIGO criteria. Study duration for each patient will be 60 ± 14 days.

**Discussion:**

It is hoped that performing hemiarch surgery using mild hypothermia (32 °C) and selective ACP will result in a 15% absolute risk reduction in the composite outcomes. The potential of this risk reduction will translate into improved patient outcomes, survival, and long-term financial savings to the health care system. In addition, the results of this trial will be used to create the first-ever guidelines for temperature management strategy during aortic surgery.

**Trial registration:**

This trial is registered on ClinicalTrials.gov with the registration number NCT02860364. Registration date August 9th, 2016.

## Background

Thoracic aortic disease (TAD) is a silent epidemic affecting 15,000 people per year, with up to 45,000 deaths per annum in the United States, making it one of the leading causes of death in people over 65 [1]. New studies have shown an increased prevalence and incidence of TAD, with surgery remaining the only viable life-saving treatment [2, 3]. However, the way in which these surgeries must be conducted carries substantial risk of morbidity and mortality.

When surgery on the aortic arch is required, the necessity of stopping systemic blood flow to provide a clear operative field places perfusion-sensitive organs, such as the brain and kidneys, at significant risk of ischemic injury. Surgery on the aortic arch was only made possible in the mid-1970s, with the introduction of deep hypothermic cardiac arrest (DHCA) by Griepp et al. [4]. The concept of deep hypothermia to reduce oxygen and metabolic requirements of hypoxic tissue is well documented [5, 6], but it is not achieved without its own adverse effects on body homeostasis and processes, including longer cardiopulmonary bypass (CPB) times, increased coagulopathy, multi-organ dysfunction, systemic inflammatory response (SIRS), endothelial dysfunction, and neuronal apoptosis [7–11]. Unfortunately, these drawbacks are intimately associated with worse neurologic (transient ischemic attack (TIA) and stroke) and renal outcomes, which often result in debilitating and lifelong illnesses for patients.

Strokes post-cardiac surgery have been shown to double the duration and costs of hospitalization, and are associated with a five- to tenfold increase in early mortality, while up to 69% of survivors suffer severe physical disability, often requiring continuing care, rehabilitation, or placement in long-term care facilities [12]. Similarly, postoperative kidney injury can result in the need for permanent dialysis, requiring on-going hospital visits and substantial increases in mortality and morbidity [13]. These postoperative complications result in significant added expenditures for the health care system. It has been estimated that the economic impact of stroke post-coronary revascularization (which carries a substantially lower risk of stroke than aortic surgery) exceeds $2 to $4 billion dollars annually worldwide [14]. Thus, it can be inferred that stroke would have a similar, if not higher, economic impact among patients undergoing aortic arch surgery.

To mitigate neurological complications, Bachet and Kazui [15, 16] developed selective anterograde cerebral perfusion (sACP). By directly cannulating the axillary or innominate artery, uninterrupted physiologic cerebral perfusion to the brain could now be maintained in patients during periods of circulatory arrest. This afforded patients almost complete neurological protection during arch surgery, effectively changing total body hypothermic circulatory arrest to isolated lower body circulatory arrest. Despite these advances in surgical technique, these procedures still carry high mortality and morbidity risks, and may be related to the adverse effects of profound hypothermia [17]. In an attempt to negate these risks, a trend towards significantly warmer core body temperatures has emerged across cardiac centers with positive outcomes being observed [8, 12, 13].

Previous retrospective studies and large meta-analyses comparing deep (14.1–20 °C) and moderate (20.1–28 °C) hypothermic cardiac arrest (MHCA) during aortic arch surgery (with sACP) have shown no differences in hospital mortality, visceral organ protection, and neurologic outcomes [8, 18, 19]. It is believed that by performing these operations at even warmer temperatures (mild hypothermia, 28.1–34 °C) the aforementioned risks associated with DHCA and MHCA can be further mitigated. Multiple case studies examining outcomes post-aortic surgery with mild hypothermia have also revealed no significant differences in mortality, renal failure, and neurological injury; even showing benefits with reduced coagulopathies and in some studies decreased permanent neurological deficits [5, 8, 20, 21].

While retrospective and uncontrolled studies are numerous and suggest the safety of warmer arch surgery [8, 17, 18], there is significant parametric variability in these studies. The optimal temperature for hypothermic circulatory arrest remains unclear and is confounded by numerous variables that are also without consensus, including site of temperature monitoring, sACP cannulation site, sACP perfusion rates, rapidity of cooling/rewarming, and the determination of outcome data [5]. Furthermore, few studies directly compare outcomes of one strategy versus another, and no prospective randomized controlled trials (RCT) exist to guide such therapy [5, 20].

This lack of evidence-based medicine has resulted in significant practice variation, with no existing guidelines for optimal perfusion strategies in aortic arch surgery. Currently, the University of Ottawa Heart Institute (OHI) performs hemiarch surgery under mild hypothermic (32 ± 2 °C) circulatory arrest and unilateral sACP (uSACP). Pilot data from our mild hypothermic patients have demonstrated excellent outcomes with lower morbidity (combined stroke, need for dialysis, and deep sternal wound infection) and decreased needs for blood products when compared to patients operated under DHCA [22]. With positive and reproducible evidence supporting mild hypothermia, there is an urgent need for a RCT to address the aforementioned questions, reduce practice variability, and produce evidence-based guidelines for perfusion strategies in aortic surgery. Thus, the purpose of this RCT is to compare clinical outcomes between patients undergoing hemiarch surgery with ACP under mild hypothermic (32 ± 1 °C) circulatory arrest versus moderate hypothermic (26 ± 1 °C) circulatory arrest.

## Methods/design

### Study hypothesis

The aim of this study is to demonstrate that circulatory arrest using mild hypothermia (32 ± 1 °C) and uSACP will result in a 15% absolute risk reduction (from 35% to 20%) — in our composite outcome of neurologic and acute kidney injury (see definition below) during aortic hemiarch surgery, when compared to moderate hypothermia (26 ± 1 °C) and uSACP.

### Study design

This trial is a prospective multi-center, single-blind two-arm RCT comparing mild versus moderate hypothermia for circulatory arrest in hemiarch surgery on a composite outcome of neurological and acute kidney injury (AKI) in 282 patients undergoing aortic hemiarch surgery with uSACP. Consenting adult patients (≥ 18 years of age) undergoing ascending aorta and hemi-arch replacement with uSACP and an anticipated circulatory arrest time of less than 20 min will be randomized 1:1 to moderate hypothermia (26 ± 1 °C, Control group) versus mild hypothermia (32 ± 1 °C, Treatment group).

### Study setting

The principal study site for this RCT will be the University of Ottawa Heart Institute (UOHI), which is a quaternary care cardiovascular center with a large thoracic aortic program, performing over 200 thoracic aortic operations per year. Other participating sites in Canada include the Foothills Medical Centre (University of Calgary), London Health Sciences Centre (Western University), and Quebec Heart and Lung Institute (University of Laval).

### Study description

#### Eligibility criteria

##### Inclusion criteria

1. Age ≥ 18 years
2. Planned unilateral or bilateral selective anterograde cerebral perfusion
3. Anticipated lower body arrest time of < 20 min
4. Able to provide written informed consent

##### Exclusion criteria

1. Surgery for aortic dissection or urgent/emergent operations
2. Total arch replacement
3. Inability to perform unilateral selective anterograde cerebral perfusion (severe axillary or innominate artery atherosclerosis/stenosis)
4. Patients with known/documented coagulopathies
5. Patients with cold agglutinin disease or those that test positive on routine preoperative screening
6. Pre-existing severe neurological impairment or inability to accurately assess neurocognitive function as determined by the operating surgeon
7. Severe carotid disease
  - Any patient with previously documented carotid stenosis of ≥ 70% (via Doppler ultrasound, magnetic resonance angiography, or computer tomography angiography) without neurological deficits
  - Carotid stenosis ≥ 50% with neurological deficits
  - Previous carotid endarterectomy or stenting
8. Patients in renal failure or currently being treated with renal replacement therapy (RRT) or estimated glomerular filtration rate (eGFR) < 30 ml/min/1.73m^2^
9. Use of an investigational drug or device at time of enrollment
10. Participation in another clinical trial which interferes with performance of this study’s procedures or assessment of outcomes

#### Enrollment

Screening will occur at the time of the initial appointment, at which time patients will be seen and assessed by the cardiac surgeon for consideration of aortic arch surgery. Patients who are booked for aortic hemiarch surgery with planned hypothermic circulatory arrest and ACP will be assessed for the aforementioned eligibility in the clinical trial. Once deemed eligible and written informed consent obtained, baseline neurological screening will be performed by trained personnel.

**Fig. 1:**
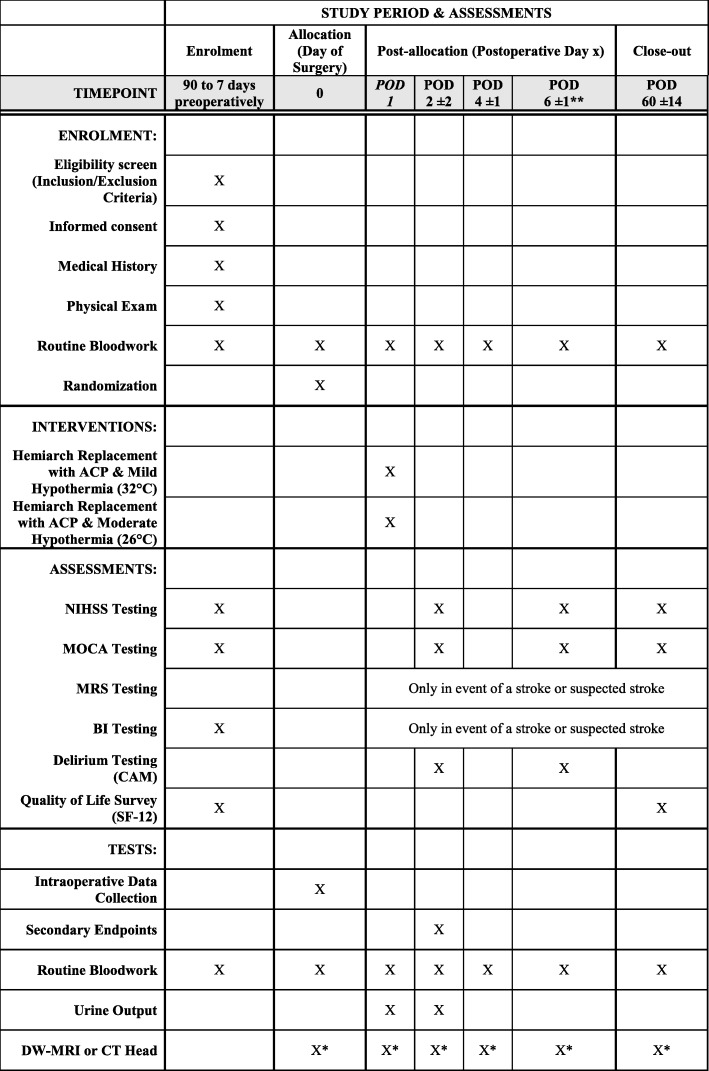
SPIRIT Figure: Summary of patient study visits and assessments. Abbreviations: POD – Postoperative Day, NIHSS – The National Institutes of Health Stroke Scale, MOCA - The Montreal Cognitive Assessment, MRS – Modified Rankin Scale, BI - Barthel Index, CAM – Confusion Assessment Method, SF-12 – Short Form Survey, DW-MRI – Diffusion-Weighted Magnetic Resonance Imaging, CT – Computer Tomography. * When clinically indicated – based on a physician assessment of the patient symptoms. ** Or prior to discharge

The following baseline information will then be collected at either this initial visit, or at the time of the preoperative anesthesia clinic visit (see SPIRIT Fig. 1):

1. Clinical information
  - Demographics—age, sex
  - HTN (hypertension)
  - DM (diabetes)
  - DLP (dyslipidemia)
  - NYHA class (New York Heart Association)
  - Aortic stenosis
    i. Graded: mild, moderate, severe
  - Aortic valve regurgitation
    i. Graded: 1 to 4+
  - CCS class (Canadian Cardiovascular Society)
  - CAD (coronary artery disease)
  - Previous history of stroke
  - Previous history of TIA (transient ischemic attack)
  - Carotid stenosis
  - Renal disease
  - PVD (peripheral vascular disease)
  - COPD (chronic obstructive pulmonary disease)
  - Pulmonary HTN
  - Smoking history
  - ETOH use (ethanol)
  - Congenital aortic disease
  - Aortic diameter
  - Euroscore II
2. Physical examination
  - Height (cm)
  - Weight (kg)
  - BMI (kg/m^2^)—body mass index
  - BSA (m^2^)—body surface area
3. Laboratory tests/investigations
  - Hb (g/L)—hemoglobin
  - Plt (× 10^9^/L)—platelet
  - INR (international normalized ratio)
  - Cr (umol/L)—creatinine
  - BUN (mmol/L)—blood urea nitrogen
  - eGFR (ml/min/1.73m^2^)—estimated glomerular filtration rate
  - HbA1C (%)—hemoglobin A1c
4. Neurocognitive testing
  - NIHSS (The National Institutes of Health Stroke Scale)
  - MOCA (Montreal Cognitive Assessment)
  - MRS (Modified Rankin Scale)—only postoperatively in event of stroke
  - BI (Barthel Index)
5. Quality of life
  - SF-12 (Short Form) survey

#### Randomization

Once study eligibility, consent, and baseline assessment are completed, subjects will be randomized using a web-based randomization tool (DACIMA 3.3.8) [23] in a 1:1 ratio on the day of surgery to either mild or moderate hypothermia strategies during aortic hemiarch surgery with uSACP. Block randomization to each treatment arm will be used across participating centres.

In order to ensure a normal distribution of kidney function among patients in both treatment arms, stratified randomization will be performed based on two eGFR ranges: 30–59 ml/min/1.73m^2^ and ≥ 60 ml/min/1.73m^2^ [24]. Randomization will also be stratified by site to reduce possible confounding from differences in standard of care practices across sites.

#### Study intervention

Subjects will be randomly allocated to one of the following two arms:

1. Mild hypothermia (32 ± 1 °C) strategy for circulatory arrest (intervention group)
2. Moderate hypothermia (26 ± 1 °C) strategy for circulatory arrest (control group)

#### Conduct of aortic hemiarch surgery and hypothermic strategy

Sequence of operation up until circulatory arrest with CPB will be at the surgeon’s discretion. All surgical procedures will be performed via median sternotomy (or upper hemi-sternotomy). During the aortic arch anastomosis, continuous, unilateral selective ACP using axillary or innominate artery cannulation will be employed. Unilateral SACP may be converted to bilateral ACP at the surgeon’s discretion if adequate cerebral flows are not achieved or if there are concerns with cerebral oximetry measurements.

Once on CPB, the patient will be cooled to a nasopharyngeal (NP) temperature of either 32 °C or 26 °C, depending on to which treatment arm the patient has been randomized. Bladder temperature and venous blood temperature will both be monitored as additional temperature sites. Unilateral SACP will only be initiated once the target temperature has been reached. ACP via the axillary or innominate artery will be commenced with target flows of 10–15 ml/kg/min with temperatures of 32 °C or 26 °C. ACP flows will be titrated to a right radial blood pressure (BP) of 60–70 mmHg. Heater/cooler temperatures are not to exceed 37.5 °C and a temperature gradient of less than 10 °C should be maintained at all times.

After completion of the aortic hemiarch replacement, CPB will be resumed and the patient re-warmed to 35 °C prior to coming off CPB, with a ≤ 1 °C temperature difference between temperature monitoring sites (NP and bladder probes).

#### Transfusion strategy

Transfusion triggers (for packed red blood cells only) will be adhered to whenever possible without compromising patient safety or center-specific transfusion protocols. Thus, a liberal transfusion threshold of Hb < 95 g/L will be used intraoperatively and in the intensive care unit (ICU), while a Hb < 85 g/L will be adhered to after transfer to the ward. These values were chosen based on the TRICS-III trial (Transfusion requirements in Cardiac Surgery III) and will be used for the duration of the patients hospital stay [25].

#### Frequency and duration of follow-up

Study participants will be followed up daily during their postoperative course in hospital, including the ICU. Intraoperative information will be collected from the anesthetic record, surgical notes, and perfusion records. Intraoperative data collection will include total operative time (skin to skin time), total CPB time, total cross-clamp time, total hypothermic cardiac arrest time, uSACP time, cooling time, re-warming time, nadir nasopharyngeal and bladder temperature, mean arterial systolic and diastolic BP, nadir hemoglobin concentration (g/L), nadir hematocrit (%), intraoperative red blood cell (RBC) transfusion (units), and highest dose/agent used for intraoperative inotrope or vasopressor support.

Postoperative data will include tabulations from the following areas:

1. Mortality
  - Death from any cause
  - Up to POD 60 ± 14
2. Neurological injury
  - TIA
  - Stroke
  - NIHSS—POD 2 ± 2, 6 ± 1 (or prior to discharge), and 60 ± 14
  - MOCA—POD 2 ± 2, 6 ± 1 (or prior to discharge), and 60 ± 14
  - MRS—only in event of postoperative stroke
  - BI—only in event of postoperative stroke
3. Delirium
  - Assessed and scored as per the Confusion Assessment Model (CAM)—POD 2 ± 2 and 6 **±** 1
4. Acute kidney injury
  - Creatinine and BUN—POD 0, 1, 2 ± 2, 4 ± 1, 6 ± 1 (or prior to discharge), and 60 ± 14
  - Urine output—up to 48 h
  - Renal replacement therapy (dialysis)
5. Prolonged mechanical ventilation
  - Mechanical ventilation times ≥ 48 h
  - Measured in hours from time of admission to the ICU
6. Coagulopathy
  - Mediastinal re-exploration for bleeding or tamponade
  - Chest tube outputs
7. Perioperative transfusions
  - Packed red blood cells (pRBCs)
  - Platelets
  - Fresh frozen platelets (FFP)
  - Cryoprecipitate
8. Postoperative myocardial infarction
  - Electrocardiogram (ECG)
  - Troponins
9. Inotropic support longer than 48 h
  - Agent (amiodarone, epinephrine, norepinephrine, dopamine, dobutamine, milrinone, vasopressin) and dose (mcg/kg/min)
10. Length of stay
  - ICU days
  - Total hospital days
11. Quality of life measures
  - SF-12—POD 60 ± 14

#### Postoperative assessment and follow-up study visits

On postoperative days 2 ± 2 days, 6 ± 1 days, and 60 ± 14 patients, will undergo neurocognitive screening by trained personnel. NIHSS and MOCA examinations will be made at each time point (or prior to discharge for time point 6 ± 1 days). In the event a neurological deficit is identified during assessment and/or based on patient symptoms (focal/global, motor/sensory loss, or prolonged delirium/agitation), neurological imaging will be obtained using computed tomography (CT) head or diffusion-weight magnetic resonance imaging (DW-MRI). MRIs will be used only if symptom onset is acute in nature requiring rapid diagnosis. Patients will also undergo MRS assessment only in the event of a postoperative stroke. BI scoring will be performed preoperatively and postoperatively only in the event of a stroke. Quality of life measures will be assessed using SF-12 health survey and obtained preoperatively and at final follow-up at 60 ± 14 days (see SPIRIT Fig. 1).

#### Primary outcome and definitions

The primary efficacy endpoint for this study will be a composite of neurologic injury and AKI that occurs at any time over the 60 ± 14 day study period. Definitions are defined in secondary outcomes below.

#### Secondary outcomes and definitions

Secondary endpoints will include:

1. Neurologic injury is divided into two major categories: temporary neurologic dysfunction (TND) or TIA and permanent neurologic dysfunction (PND) or stroke
  - TND or TIA—neurologic symptoms lasting < 24 h and without evidence of infarction
    i. Neurological imaging has to be normal with resolution of all symptoms within 24 h
  - PND or stroke—presence of either new focal (embolic stroke) or global (diffuse coma) deficits which persist for longer than 72 h
    i. Positive radiographic evidence of infarction in the appropriate territory

In the event of a suspected neurological injury the clinical team will be alerted and the study neurologists will be consulted. Neurologic injury will be quantified using a combination of validated cognitive screening tests and neurologic imaging. Screening tests will be performed preoperatively and on postoperative days 2 ± 2, 6 ± 1 (or prior to discharge), and 60 ± 14 by trained personnel (study coordinator, study neurologist, study nurse practitioner). Note both BI and MRS scores will only be performed postoperatively in the event of a postoperative stroke and will occur at the same times as the follow up NIHSS/MOCA testing. These points in time have been chosen based on previous studies [26] that actively monitor for stroke post-cardiac surgery, as well as to allow for long-term follow up of patients post-stroke. Screening tests include:

c. The National Institutes of Health Stroke Scale (NIHSS)—highly predictive of hospital disposition and long-term stroke outcomes. Scores range from 0 to 42. It has been shown for each one-point increase in NIHSS, the likelihood of going home is significantly reduced [27–29]
  i. 0 = no stroke
  ii. 1–4 = minor stroke
  iii. 5–15 = moderate stroke
  iv. 15–20 = moderate/severe stroke
  v. 21–42 = severe stroke
  vi. Study neurologists following serial NIHSS scores will determine whether there was a change in examination from previous exams and whether this change was because of a suspected stroke
    1. For the purpose of this study, severe strokes are defined as NIHSS ≥ 10 [26]
    2. Clinically significant strokes are defined as a change in NIHSS ≥ 4
      - This is based on data which show NIHSS scores < 6 indicate a strong likelihood of hospital discharge, with good recovery, and no long-term deficits [28, 30]
d. The Montreal Cognitive Assessment (MOCA)—highly sensitive in detecting executive dysfunction. Scores range from 0 to 30 [31]
  i. Scores > 26—no cognitive impairment (normal exam)
  ii. Scores < 26—mild cognitive impairment
e. Modified Rankin Scale (MRS)—only to be used in the event of a postoperative stroke. The MRS is a reliable and reproducible scoring method for the assessment of deficits post-stroke in patients. Scores range from 0 to 6 [32, 33]
  i. 0, no symptoms
  ii. 1, no significant disability: able to carry out all usual activities, despite some symptoms
  iii. 2, slight disability: able to look after own affairs without assistance, but unable to carry out all previous activities
  iv. 3, moderate disability: requires some help, but able to walk unassisted.
  v. 4, moderately severe disability: unable to attend to own bodily needs without assistance, and unable to walk unassisted
  vi. 5, severe disability: requires constant nursing care and attention, bedridden, incontinent
  vii. 6, dead
f. Barthel Index of Activities of Daily Living (BI)—the BI is formed by several disability indexes and is a reliable and reproducible scoring method for the assessment of activities of daily living (ADLs) post-stroke in patients. Scores range from 0 to 20 [34–36]
  i. Lower scores indicate increased disability
  ii. When used to measure improvement after rehabilitation, changes of more than two points in the total score reflect probable genuine change
  iii. Change on one item from fully dependent to independent are also likely to be genuine

2. Incidence of acute kidney injury (AKI)
  - Postoperative AKI will be assessed using the KDIGO criteria [37–39] and are summarized in Table 1
  - Creatinine measurements will serve as the main indicator for assessing AKI
  - All those with KDIGO stage 1 or higher AKI will be considered to have sustained postoperative AKI
  - Creatinine measurements will be performed with routine bloodwork on POD 0, 1, 2 ± 2, 4 ± 1, 6 ± 1, and 60 ± 14 days
  - Urine output for the diagnosis of AKI will only be measured in the immediate postoperative period (48 h)
  - Blood urea nitrogen (BUN) levels will also be obtained on the same days

**Table 1:** KDIGO classification of AKI

Stage	Serum creatinine	Urine output
1	1.5–1.9 times baseline or ≥ 0.3 mg/dl (26.5 μmol/l) increase	< 0.5 ml/kg/h for 6–12 h
2	2.0–2.9 times baseline	< 0.5 ml/kg/h for ≥12 h
3	3.0 times baseline or Increase in serum creatinine to ≥ 4 mg/dl (353.6 μmol/l) or initiation of renal replacement therapy	< 0.3 ml/kg/hr. for ≥24 h or Anuria for ≥ 12 h
3. Incidence of delirium
  - Delirium—reversible postoperative delirium lasting more than 48 h without localizing signs
    i. Delirium assessment will be performed using CAM
      1. For a diagnosis of delirium by CAM, the patient must display:
        - Presence of acute onset and fluctuating course AND
        - Inattention
      2. And either one of:
        - Disorganized thinking OR
        - Altered level of consciousness
    ii. Assessments will be performed on POD 2 ± 2 and 6 **±** 1
4. Death
  - All cause postoperative 60-day or in-hospital mortality
5. Cardiopulmonary bypass (CPB) time (minutes)
6. Bleeding rates to qualify for mediastinal re-exploration
  - Indication for exploration defined as postoperative mediastinal bleeding of [40, 41]:
    i. 500 ml in any one hour
    ii. 1000 ml in any 4-h period
    iii. Or at surgeons discretion
7. Incidence and quantity (number of units/patient) of perioperative blood transfusions
  - Defined as all intraoperative and postoperative transfusions up to POD 7 or discharge (whichever comes first)
  - The transfusion of packed red blood cells (pRBCs) will adhere to the previously defined transfusion trigger strategy whenever possible without compromising patient safety
    i. A liberal transfusion threshold of Hb < 95 g/L will be used intraoperatively and in the ICU, while a Hb < 85 g/L will be adhered to after transfer to the ward
  - Number of platelets, fresh frozen plasma (FFP), and cryoprecipitate will also be tabulated for the same time period
    i. No transfusion triggers given and left to discretion of caring physician
8. Prolonged mechanical ventilation
  - Defined as those requiring ≥ 48 h of mechanical ventilation
  - Measured in hours from time of admission to the ICU
9. Perioperative myocardial infarction
  - Clinically diagnosed using a combination of electrocardiographic (new Q wave on 12 lead ECG) and biochemical (troponin I > 45 ng/L) markers or both
10. Length of stay
  - ICU hours
  - Hospital days
11. Quality of life (SF-12) survey
  - A measure of perceived health (health-related quality of life) that describes the degree of general physical health status and mental health distress [42]
  - Will be assessed preoperatively and on POD 60 ± 14

### Statistical consideration

In a prospective contemporary study of patients over the age of 65 years undergoing aortic valve replacement, Floyd and colleagues observed an incidence of clinical stroke of 17% using similar assessment modalities to those proposed in this trial [26]. Interestingly, clinically documented strokes in the Society of Thoracic Surgeons (STS) adult cardiac surgery database in the same patients were only 7%, suggesting that careful and systematic documentation can reveal a ~ 2.5-fold higher incidence of neurologic injury than routine clinical evaluation. Furthermore, this study found increasing CPB to be an important risk factor for neurologic injury [26].

In patients undergoing aortic arch surgery, the incidence of neurologic injury is expected to be higher due to a variety of reasons, including longer CPB times, manipulation of the aortic arch and branch vessels for cannulation or clamping, and injury associated with hypothermia and re-warming. In a large retrospective study of over 45,000 patients undergoing proximal aortic arch replacement surgery, Williams et al. found a clinical stroke rate of ~ 6.62%.[43] Notably, the authors did not include patients who may have suffered TIAs or other temporary neurologic dysfunction. Their data were also obtained from the STS database, with no prospective or systematic neurologic evaluation. Thus, for the purpose of this RCT, we hypothesize that patients in the moderate hypothermia group will experience a 15–20% incidence of neurologic injury.

With respect to the incidence of AKI, previously reported rates of stage 1 AKI in patients undergoing cardiac surgery by Boodhwani and colleagues have revealed an incidence of around 22.35% based on the Acute Kidney Injury Network (AKIN) criteria for staging AKI [44]. Their study population was a mixed surgical population with infrequent procedures such as heart transplantation, ventricular assist device placement, and complex congenital abnormality repair being excluded.

This RCT will use the newer KDIGO criteria for assessing AKI, which identifies significantly more AKI in patients than both the Risk, Injury, Failure, Loss of kidney function, and End-stage kidney disease (RIFLE) and AKIN criteria [38, 45]. Specifically, KDIGO criteria have been shown to have a greater sensitivity in diagnosing AKI when compared to RIFLE and AKIN (51% versus 46.9% for RIFLE, *p* <  0.01 and 38.4% for AKIN, *p* <  0.001) [45]. It is also expected that the incidence of AKI will be higher in this study based on the type of surgery being performed, which necessitates a period of lower body ischemia time. Thus, we hypothesize a 25% incidence of KDIGO stage 1 AKI for this trial.

#### Sample size

Taking into consideration the aforementioned incidence rates of both neurologic injury and AKI, a composite outcome of ~ 35% should represent the incidence of these injuries in patients undergoing hemiarch surgery using traditional methods of hypothermia. This accounts for a 5–10% overlap that will likely exist, where a patient will suffer both types of injuries.

We hypothesize that circulatory arrest using mild hypothermia (32 °C) and uSACP during aortic hemiarch surgery will result in a 15% absolute risk reduction in composite outcomes (neurologic injury and AKI) from 35% to 20%. With an alpha (type 1 error) of 0.05 and power of 80%, and a 5% loss to follow-up, approximately 141 subjects will be needed in each group for a total of a 282 patients.

#### Clinical significance

As mentioned, strokes post-cardiac surgery have been shown to double the duration and costs of hospitalization, and are associated with increased mortality and severe physical disability [12]. Similarly, kidney injury resulting in end-stage renal disease is a major health problem due to its high morbidity and mortality, as well as having social and financial implications [46]. These post-operative complications result in significant lifestyle morbidity, as well as added expenditures to the health care system.

With an absolute risk reduction (ARR) of 15%, the relative risk (RR) and RR reduction (RRR) would be 57% and 43%, respectively, suggesting the proposed intervention carries benefit, and has a medium-sized “relative” effect, with only seven patients needing to be treated with the mild hypothermic strategy to avoid an adverse event. Considering the proposed intervention adds no extra costs, with temperature being the only variable manipulated in the normal standard of care for these patients, the proposed intervention potentially carries both a significant and meaningful benefit to patients while reducing health care expenditure.

#### Statistical analysis

The trial will be analyzed on a true intention to treat (ITT) basis, including all trial participants who were randomized regardless of adherence to treatment protocol, including those who are lost to follow-up or may have died. Patient crossover will be tracked and reported with the final trial results. In addition, a ‘modified ITT’ analysis will be performed (excluding patients not assessed for the primary outcome or lost to follow-up and/or early death) to account for potential biases introduced with ITT analyses.

The primary endpoint of our composite outcome will be evaluated using a chi squared test (or Fisher’s exact test if cell count is < 5 in any cell). Categorical secondary endpoints will be analyzed in the same method, while continuous secondary endpoints will be analyzed using Students *t*-test (or Wilcoxon rank sum test if the data are not normally distributed). Continuous variables will be reported as mean ± standard deviation or median (interquartile range [IQR]) for non-normally distributed variables. Categorical variables will be reported as counts and percentages. As the primary outcome is a composite of both neurologic and AKI, secondary outcome analysis will pay particular attention to individual assessment of these indices for treatment effect and positive correlation.

Exploratory multivariable logistic regression analysis will be performed to determine risk factors for neurologic and renal complications post-operatively. A *p* value of < 0.05 will be considered statistically significant. Multivariable analysis will include variables with *p* <  0.20 in univariate analysis in addition to clinically significant variables.

Once data collection is complete for all study participants, a statistical analysis plan (SAP) will be created prior to data analysis and release of results. As the principle statistician involved in this trial, Dr. George Wells (PhD) will primarily be responsible for this analysis. Dr. Wells is the Director of the Cardiovascular Research Methods Centre at the UOHI and Professor in the School of Epidemiology, Public Health and Preventive Medicine at the University of Ottawa.

#### Data collection—case report forms

Data collection will be completed by authorized study personnel designated by the site investigator. Appropriate training will be completed with the site investigator and all authorized personnel prior to the study being initiated. Data collection started on paper; however, an electronic data collection system has been developed and is now used as the primary data collection method for all sites.

#### Monitoring and auditing

Monitoring of study compliance and data collection from other sites will be done by the clinical nursing coordinator at the primary trial site – University of Ottawa Heart Institute. This will involve regular follow-up phone conversations as well as on site trial visits.

A Data Safety Monitoring Board (DSMB) will be assembled to assess the ongoing conduct of the trial. The DSMB will have at least three members with sufficient expertise in aortic surgery, clinical research methods, and statistics. The DSMB will meet twice per year, most often by teleconference, and will provide a summary report to the study team, who will in turn submit it to the Research Ethics Board (REB) and Ottawa Heart Institute Research Corporation (OHIRC) research administration before the due date. The terms of reference for the DSMB have been drafted following OHIRC’s template, and will be stored with the study regulatory files.

## Discussion

The principal site for this trial will be the University of Ottawa Heart Institute (UOHI), which is a quaternary care cardiovascular center with a large thoracic aortic program, performing over 200 thoracic aortic operations per year. We perform approximately 40 operations per year that meet the aforementioned recruitment criteria of this trial. Assuming an 80% recruitment rate, we anticipate that we will be able to randomize approximately 30 patients per year into this study at the UOHI.

Three other participating centers with similar volumes of aortic operations per year will be taking part in this trial. The centers include the Foothills Medical Centre (University of Calgary), London Health Sciences Centre (Western University), and Quebec Heart and Lung Institute (University of Laval). We anticipate these three centers to randomize approximately 15–20 patients per year, for an additional 45–60 patient enrollment rate per year. Total enrollment rate for all centers will be 75–90 patients per year.

### Non-compliance, loss to follow-up, early termination, concomitant medications, and procedures

The proposed intervention is strictly intraoperative. Both pre- and postoperative courses will follow institutional standard practices of care. As such, non-compliance is not expected to be a significant issue in this trial.

Loss to follow-up is expected to be low as both pre- and postoperative courses are as per normal standard of care. This study will follow participants for neurological screening up to 60 ± 14 days postoperatively, which may translate to only one additional patient visit that falls outside of the normal standard of care. Thus, we anticipate the rate of loss to follow-up will be low at around 5%. Loss to follow-up will only occur after multiple attempts have been made to contact the study participant by telephone, email, and/or registered mail for final in-hospital assessment.

All subjects are free to withdraw from participating in the study at any time and for any reason. Additionally, subjects may be excluded from the study for specific reasons, including ineligibility, change in preoperative diagnosis and/or condition, or non-compliance with study follow-up activities.

There are no restrictions on medication use or food intake for this study. All medications, including over-the-counter (OTC) medications, herbal, and natural remedies will be recorded on the study participant’s medication profile at pre-op and prior to discharge from hospital, as well as during follow-up visits. As such, subjects will continue to receive all usual medications, rehabilitation, procedures, and interventions as prescribed or recommended by his/her health care providers.

### Adverse event reporting

An adverse event (AE) reporting form has been created to collect, assess, report, and manage the occurrence of adverse events that may impact the trial. The form collects date of event, date of study staff notification, as well as the participant’s unique identifier. A description of the event, subsequent interventions/treatments, event outcome, and classification as serious and/or unexpected is also included on the form. All AEs are then assessed by the principal investigator for their relationship to study treatment and classified on the AE form as either unlikely related, possibly related, probably related, or related (see Additional file 1 for the adverse events reporting form).

### Blinding, clinical/study staff, and participant confidentiality

Knowledge of the patient’s treatment arm has the potential to bias postoperative care, especially with respect to transfusion strategies and decision-making when seeking neurodiagnostic imaging for possible deficits. In order to limit these biases, patient temperatures and CPB times will be redacted from the relevant operative room (OR) records shortly after surgery. This will be performed by a dedicated un-blinded assistant at all study sites. Operating surgeons will also be instructed not to include CPB time or patient temperatures in their dictated operative notes. A copy of the un-redacted OR records will be placed in a sealed envelope and placed with the patient’s chart. The envelope will have the name and contact information of the principal investigator (PI), with instructions to open the envelope only if there is a medical need to know the patients intraoperative temperature or CPB time.

It will be suggested that clinical staff speak directly with the PI prior to opening this envelope. Upon the patient’s discharge, the envelopes will be stored in a locked file cabinet in the Clinical Research Office. As the envelopes contain identifiable information, they will not be stored with the patient’s study file.

As the administration and/or scoring of both the neurocognitive tests and the quality of life questionnaire may be prone to bias, any study staff administering neurocognitive tests and quality of life questionnaires will be blinded to the patient’s group assignment at all times.

All study-related information, including case report forms (CRFs), evaluation forms, and consultation reports, will be kept strictly confidential. All records will be kept in a secure, locked location and only research staff will have access to the records. Subjects will be identified only by means of a study ID number specific to each subject. All computerized databases will identify subjects only by study ID numbers and will be maintained on secure hospital servers.

A password-protected master list containing both the study ID and the patient’s name and contact information will be maintained on a secure hospital server, in a folder accessible only by study staff.

## Trial status

This trial is registered on ClinicalTrials.gov (https://clinicaltrials.gov/) with the registration number NCT02860364. The trial was registered on August 9^th^, 2016. The most up to date protocol version is “Protocol Version 13 – November 20^th^ 2018”. Recruitment started March 1^st^, 2018. Date of recruitment completion is anticipated to be August 1^st^, 2021.

## Supplementary information

**Additional file 1.** Adverse event form. (PDF 663 kb)

**Additional file 2.** Participant informed consent forms. (PDF 919 kb)

**Additional file 3.** SPIRIT 2013 Checklist: Recommended items to address in a clinical trial protocol and related documents. (DOC 123 kb)

## Data Availability

Not applicable, as this is a study protocol.

## Abbreviations

ACP: Antegrade cerebral perfusion
ADL: Activity of daily living
AKI: Acute kidney injury
AKIN: Acute Kidney Injury Network
BI: Barthel Index
BMI: Body mass index
BSA: Body surface area
BUN: Blood urea nitrogen
CAD: Coronary artery disease
CAM: Confusion assessment method
CCS: Canadian Cardiovascular Society
COPD: Chronic obstructive pulmonary disease
CPB: Cardiopulmonary bypass
Cr: Creatinine
CTA: Computer tomographic angiography
DHCA: Deep hypothermic cardiac arrest
DLP: Dyslipidemia
DM: Diabetes
DSMB: Data Safety Monitoring Board
eGFR: Estimated glomerular filtration rate
ETOH: Ethanol
Hb: Hemoglobin
HTN: Hypertension
ICU: Intensive care unit
INR: International normalized ratio
ITT: Intention to treat
KDIGO: Kidney Disease Improving Global Outcomes
LOS: Length of stay
MHCA: Moderate hypothermic cardiac arrest
MOCA: Montreal Cognitive Assessment
MRA: Magnetic resonance angiography
MRS: Modified Rankin Scale
NIHSS: National Institutes of Health Stroke Scale
NYHA: New York Heart Association
OHRIC: Ottawa Heart Institute Research Corporation
Plt: Platelet
PND: Permanent neurologic dysfunction
POD: Post-operative day
pRBC: Packed red blood cells
PVD: Peripheral vascular disease
RCT: Randomized control trial
REB: Research ethics board
sACP: Selective antegrade cerebral perfusion
SIRS: Systemic inflammatory response
TAD: Thoracic aortic disease
TIA: Transient ischemic attack
TND: Temporary neurologic dysfunction
US: Ultrasound
uSACP: Unilateral selective antegrade cerebral Perfusion
