# Supplementary material for: COMMENCE trial (Comparing hypOtherMic teMperaturEs duriNg hemiarCh surgEry): a randomized controlled trial of mild vs moderate hypothermia on patient outcomes in aortic hemiarch surgery with anterograde cerebral perfusion
Source: Trials. 2019 Dec 9;20:691. doi: 10.1186/s13063-019-3713-9 (PMC6902484; doi:10.1186/s13063-019-3713-9)
Supplement: Supplementary file 1 — Additional file 1. Adverse event form. (PDF 663 kb) [file 13063_2019_3713_MOESM1_ESM.pdf]

COMMENCE: Adverse Event Form

Event Number# ___________ Date of event: / /

Date study staff aware of event: / /

MACE Event List

❍ Death from Cardiovascular Cause	*Death from cardiovascular cause is defined as any death for which there is no clearly documented nonvascular cause*
❍ Peri-operative Myocardial Infarction	*Peri-operative Myocardial Infarction is defined as cTn measurement ≥ 10 × 99th percentile upper reference limit (URL) during first 48 hours following CABG, with at least 1 of the following: A) with new pathologic Q waves or new LBBB (Q-wave MI); B) angiographic evidence of new graft or native coronary artery occlusion; or C) imaging evidence of new loss of viable myocardium or new regional wall motion abnormality.*
❍ Non-perioperative Myocardial Infarction	*defined as a detection of rise and/or fall of cTn with at least one value above the 99th percentile of the URL more than 48 hours after the surgery, together with evidence of myocardial ischemia with at least 1 of the following: A) symptoms of ischemia; B) ECG changes indicative of new ischemia (new ST-T changes or new LBBB); C) development of pathologic Q waves in the ECG; D) Imaging evidence of new loss of viable myocardium or new regional wall motion abnormality; or E) identification of an intracoronary thrombus by angiography or autopsy.*
❍ Cerebrovascular Accident	*Stroke is defined as A) new, acute focal neurological deficit thought to be of vascular origin with signs or symptoms lasting >24 hours and confirmed by a neurologist; B) new, focal neurological deficit lasting > 24 hours with imaging evidence of cerebral infarction or intracerebral hemorrhage; C) non-focal encephalopathy lasting >24 hours with imaging evidence of cerebral infarction or intracerebral hemorrhage.*
❍ Coronary Ischemia	*Recurrent Ischemia after surgery is defined as re-hospitalization for classified unstable angina*
❍ Target vessel revascularization	*defined as a new CABG procedure or PCI associated with documented ischemia by stress testing (ECG, echocardiography, or nuclear testing), graft failure, new culprit lesion (≥70% luminal stenosis), or determined by the patient's physician to be clinically indicated.*
❍ Conversion to sternotomy	*For patients undergoing MICS CABG, the need for doing a sternotomy for any reason (including completing anastomosis, control bleeding, improve hemodynamics).*
Re-opening for bleeding	*defined as requirement to return to OR for re-opening of sternotomy or MICS CABG incision for any reason, within the same hospital admission as the completed procedure.*
❍ Other	*Adverse event whether serious, not serious, related not related, expected, not expected that has an untoward medical occurrence or unfavorable and unintended sign in a subject per investigators decision.*

[Continued on next page]

[Continued from previous page]

Event:

EVENT DESCRIPTION

Event Number# ____________

Description of Event (attach source documents as relevant)

Interventions/Treatments

Event outcome: ❍Resolved – date _______ ❍Death – date _________ ❍Ongoing

Is/was the event serious? $_0$❍ N $_1$❍ Y

Is/was the event unexpected? $_0$❍ N $_1$❍ Y

Assessment by Principal Investigator

Relationship to study treatment

○ **Unlikely Related -** no temporal association or the cause of the event has been identified, or the drug, biological or device cannot be implicated

○ **Possibly Related** - temporal association, but other etiologies are likely to be the cause; however involvement of the drug, biological, or device cannot be excluded.

○ **Probably Related** - temporal association and other etiologies are possible but unlikely

○ **Related** - definite association to drug, biological or device is certain

If event is serious, unexpected, and possibly/probably related to study treatment, expedited reporting to Sponsor, REB and appropriate regulatory authorities is required.

____ ___________________________

Signature of Investigator assessing relationship Date (dd/mmm/yyyy)
