# Supplementary material for: COMMENCE trial (Comparing hypOtherMic teMperaturEs duriNg hemiarCh surgEry): a randomized controlled trial of mild vs moderate hypothermia on patient outcomes in aortic hemiarch surgery with anterograde cerebral perfusion
Source: Trials. 2019 Dec 9;20:691. doi: 10.1186/s13063-019-3713-9 (PMC6902484; doi:10.1186/s13063-019-3713-9)
Supplement: Supplementary file 2 — Additional file 2. Participant informed consent forms. (PDF 919 kb) [file 13063_2019_3713_MOESM2_ESM.pdf]

The Ottawa Hospital | L'Hôpital d'Ottawa
RESEARCH INSTITUTE | INSTITUT DE RECHERCHE

uOttawa

UNIVERSITY OF OTTAWA HEART INSTITUTE
INSTITUT DE CARDIOLOGIE DE L'UNIVERSITÉ D'OTTAWA

PARTICIPANT INFORMED CONSENT FORM

Title of Study: COMMENCE Trial – Comparing hypOtherMic teMperaturEs duriNg hemiarCh surgEry. A Randomized Controlled Trial of Mild vs Moderate Hypothermia on Patient Outcomes in Aortic Hemiarch Surgery with Anterograde Cerebral Perfusion

OHSN-REB Protocol No: 20160408

Local Site Principal Investigator (PI): Dr. Munir Boodhwani – 613-696-7295

Sponsor: Ottawa Heart Institute Research Corporation (OHIRC)

Funding Agency: Internal funding, Division of Cardiac Surgery, OHIRC

Participation in this study is voluntary. Please read this Participant Informed Consent Form carefully before you decide if you would like to participate. Ask the study doctor and study team as many questions as you like. We encourage you to discuss your options with family, friends or your healthcare team.

Why am I being given this form?

You are being asked to participate in this research study because you are a patient that is going to have surgery on the hemiarch portion of your aorta.

Why is this study being done?

Surgery of the aorta can be technically difficult, and cardiac surgeons have a number of ways to help reduce the risks for patients. In some aortic surgeries, the surgeon needs to stop the patient's heart for a short period of time. While the heart is stopped, the surgical team uses machines to ensure that blood is pumped where it is needed in the patient's body, most importantly to the brain.

In order to protect the brain and other organs while the heart is stopped, the surgical team carefully lowers the patient's body temperature to cause hypothermia. This type of controlled hypothermia helps to slow down the regular activities of the body's cells, which helps protect brain cells from damage while the heart is stopped. Unfortunately, lowering a person's body temperature has risks as well. Patients may have bleeding problems, their organs may not recover completely, they may experience damage to their brain, and they can experience a serious medical condition called Systemic Inflammatory Response Syndrome. Lowering the body

temperature also means that the heart may be stopped for longer, as they have to warm the body up slowly before the heart can start again.

The purpose of this study is to compare moderate hypothermia (26°C) to mild hypothermia (32°C) during surgery on the hemiarch portion of the aorta. The research team is most interested in whether using milder hypothermia will reduce the chance of patients experiencing neurological or kidney injury. We will try to find out if the different temperatures make patients more or less likely to have bleeding events, inflammation, neurologic problems, or dysfunction of the internal organs. We want to carefully look at both temperatures to try to find which temperature is cold enough to protect the brain and organs, but warm enough to reduce the complications of hypothermia and the length of time the heart is stopped.

The two different temperatures are both used during aortic surgeries at hospitals across Canada. This study is taking place at a number of sites across North America. We estimate that 282 participants will be enrolled in the study, including 80 participants from the University of Ottawa Heart Institute.

How is the study designed?

This is a study with two study groups. One group will have moderate hypothermia (26° C) during their aortic hemiarch surgery and the other group will have mild hypothermia (32°C). The study group you are in will be decided randomly. This means that you are put into a group by chance similar to flipping a coin.

This study will be blinded, which means that you will not be told which group you are in. Some members of the study team will know what group you are in, but some members of the study team and the clinical staff that take care of you after surgery will not. Blinding helps to remove any bias or pre-conceived notions from affecting the outcome of the study. You may be told once the study is finished. If the clinical staff taking care of you after surgery needs to know what group you are in, they will be able to find out very quickly.

This study will include a sub-group to look for evidence of "silent strokes". These are strokes that do not produce any symptoms and are only seen by post-surgery magnetic resonance imaging (MRI). A subset of 58 participants from each study group will be randomly chosen to have an MRI to look for these silent strokes.

What is expected of me?

If you decide to take part in the study, you will see someone from the study team before your surgery for a screening visit. This visit will help determine whether will be eligible to be in the study. On the day of surgery, you will be randomly assigned to have either mild or moderate hypothermia during your surgery.

A member of the research team will visit you while you are recovering in the hospital after surgery, and you will be asked to return for one visit between 30 and 90 days after your surgery. This visit will be at the same time as your post-operative visit with your surgeon.

Study Visits and Procedures. Boxes marked with an X show what will happen at each visit.

Visit	Screening	Day of Surgery	Post Op Day 0 -6	60 +/-30 days after surgery
Length of time needed	1 hour	surgery	1 hour	1 hour
Medical history, medications, current health, inclusion/exclusion	X		X	X
Informed Consent	X			
Questionnaires	X		X	X
Randomization/Intervention		X		
Routine Bloodwork			X	
MRI*			X	

<u>Questionnaires</u> – The study team will use 1 questionnaire to measure your health-related quality of life, or the way that your health affects your day-to-day activities. The study team will use 4 questionnaires to measure your neurocognitive functions such as memory, the ability to follow directions, and attention. You will be asked to complete one or more these questionnaires once before your surgery, at days 2 and 6 after your surgery, and at the 30- and 90-day follow up visits. These questionnaires will take approximately 25 minutes to complete. You may skip any questions that make you uncomfortable or that you do not wish to answer.

<u>*MRI (magnetic resonance imaging) subgroup</u> – After you are enrolled in the study, you may be randomly selected to complete an MRI to scan your brain for evidence of stroke. No symptoms are needed to be selected for the MRI subgroup. 58 patients in each study arm will be randomly chosen for an MRI. The MRI will take approximately 90 minutes.

<u>How long will I be involved in the study?</u>
The entire study will last approximately 5 years. Your participation in the study will last approximately 3 months. Over this time, you will be required to visit the University of Ottawa Heart Institute 2 times for the study. These visits will be completed while you are already coming to the University of Ottawa Heart Institute for visits with your doctor. .

Your participation in the study may be stopped for any of the following reasons:
- The study doctor feels it is in your best interest.
- You do not follow the study staff’s instructions.

<u>What are the potential risks I may experience?</u>
This study procedure has risks, as most procedures do. However, there is always a chance of risks that we do not know about. The risks we know about are:

<u>Hypothermia during aortic cardiac surgery</u>
We do not know the difference in risks between mild versus moderate hypothermia during aortic surgery. Studies of patients having aortic surgery with hypothermia have shown approximately a 7% risk of stroke, an 8% risk of kidney failure, and an overall risk of death during and after surgery of about 3% These risks are not specific to this study procedure, they are the risks of aortic surgery with hypothermia in general.

Questionnaires:
You might find the interviews and questionnaires to be long. You might not like all of the questions that you are asked. You do not have to answer any questions that make you uncomfortable.

MRI:
Most MRI exams are painless. Some patients find it uncomfortable to remain still during the imaging. Your body may feel slightly warm. You will hear the loud tapping or thumping as the machine takes the images. A doctor will be able to see and hear you at all times, so if you feel any discomfort you can let them know. In some cases, a contrast dye may be injected into your arm. This may cause swelling or bruising, and irritation at the site of injection. You may have a metallic taste in your mouth after the injection. In rare cases the contrast dye may cause an allergic reaction such as hives or itchy eyes, or other reactions.

Risks of Insurability:
We will take all reasonable steps to keep your research information confidential. Should someone not involved in the research find out that you took part in this research study, or if you choose to share your results (if they are provided to you), there is a possibility that this could affect your insurability under certain policies of insurance, depending on the exclusions in such policies.

Can I expect to benefit from participating in this research study?
You may not receive any direct benefit from your participation in this study. Your participation may allow the researchers to better understand whether mild or moderate hypothermia is better for patients. This may benefit future patients.

This study will select by chance which treatment you will receive. Participants in one arm of this study may do better or worse than participants in the other arm.

Do I have to participate? What alternatives do I have?
You can choose not to participate in this study. If you choose not to participate, you will receive the standard care provided to all patients undergoing aortic hemiarch surgery, which may include either mild or moderate hypothermia. Your study doctor will discuss these options with you.

Your participation in this study is voluntary. You may decide not to be in this study, or to be in the study now, and then change your mind later without affecting the medical care, education, or other services to which you are entitled or are presently receiving at this institution.

If I agree now, can I change my mind and withdraw later?
You may withdraw from the study at any time without any impact on your current or future care at this institution.

- If you decide to stop your study participation in the study, you should contact the study doctor or the study team first. They will discuss the related issues or possible safety concerns for you.
- You may also choose to discontinue your participation in the study. However, a final visit(s) may need to be completed to ensure your safety and well-being.
- If you withdraw your consent, the study team will no longer collect your personal health information for research purposes, unless it is needed for review of safety. The study team may still use the information they have collected up to that point. If you don't want any of your data to be used, you should speak with the study doctor.

What compensation will I receive if I am injured or become ill in this study?
In the event of a study-related injury or illness, you will be provided with appropriate medical treatment and care. Financial compensation for lost wages, disability or discomfort due to an injury or illness is not generally available. You are not waiving any of your legal rights by agreeing to participate in this study. The study doctor and the University of Ottawa Heart Institute still have their legal and professional responsibilities.

Will I be paid for my participation or will there be any additional costs to me?
The cost of parking/transportation will be paid for any extra visits related to the study.

How is my personal information being protected?
- If you decide to participate in this study, the investigator(s) and study staff will look at your personal health information and collect only the information they need for this study. "Personal health information" is health information about you that could identify you because it includes information such as your name, address, telephone number, date of birth, new and existing medical records, or the types, dates and results of various tests and procedures.
- Information that identifies you will be released only if it is required by law.
- All information collected during your participation in this study will be identified with a unique study number (for example participant # AB01), and will not contain information that identifies you.
- A Master List provides the link between your identifying information and the coded study number. This list will only be available to Dr. Boodhwani and his staff and will not leave this site.
- The Master List and coded study records will be stored securely.
- For audit purposes only, your original medical records may be reviewed under the supervision of Dr. Boodhwani's staff by representatives from:
    - The Ottawa Health Science Network Research Ethics Board (OHSN-REB), and the Ottawa Heart Institute Research Corporation.
- You will not be identified in any publications or presentations resulting from this study.
- Research records will be kept for 10 years, as required by the OHSN-REB.
    - At the end of the storage time, all paper records will be shredded and all electronic records will be securely deleted.

A description of this clinical trial will be available on http://www.ClinicalTrials.gov. This Web site will not include information that can identify you. At most, the Web site will include a summary of the results. You can search this Web site at any time.

This research study can be found on the above listed website by using the clinical trial registration number NCT02860364.

Do the investigators have any conflicts of interest?
There are no conflicts of interest to declare related to this study.

What are my responsibilities as a study participant?
It is important to remember the following things during this study:

- Ask the study team if you have any questions or concerns.
- Tell your study team if anything about your health has changed.
- Call the study doctor if you experience any side effects, even if you are unsure whether it has anything to do with this study.

Will I be informed about any new information that might affect my decision to continue participating?
You will be told in a timely fashion of any new findings during the study that could affect your willingness to continue in the study. You may be asked to sign a new consent form.

Who do I contact if I have any further questions?
If you have any questions about this study, or if you feel that you have experienced a study-related injury or illness, please contact Dr. Boodhwani at 613-696-7295 or the study staff at 613-696-7000 extension 18329.

The Ottawa Health Science Network Research Ethics Board (OHSN-REB) has reviewed this protocol. The Board considers the ethical aspects of all research studies involving human participants at the University of Ottawa Heart Institute. If you have any questions about your rights as a study participant, you may contact the Chairperson at 613-798-5555, extension 16719.

COMMENCE Trial – Comparing hypOtherMic teMperaturEs duriNg hemiarCh surgEry. A Randomized Controlled Trial of Mild vs Moderate Hypothermia on Patient Outcomes in Aortic Hemiarch Surgery with Anterograde Cerebral Perfusion <u>(OHSN-REB# 20160408)</u>

<u>Consent to Participate in Research</u>

- I understand that I am being asked to participate in a research study about different hypothermia temperatures used during aortic hemiarch surgery.
- This study was explained to me by ____________________________.
- I have read, or someone has read to me, each page of this Participant Informed Consent Form.
- All of my questions have been answered to my satisfaction.
- If I decide later that I would like to withdraw my participation and/or consent from the study, I can do so at any time.
- I voluntarily agree to participate in this study.
- I will be given a copy of this signed Participant Informed Consent Form.

______________________________	______________________________	____________________
Participant's Printed Name	Participant's Signature	Date

<u>Investigator or Delegate Statement</u>

I have carefully explained the study to the study participant. To the best of my knowledge, the participant understands the nature, demands, risks and benefits involved in taking part in this study.

______________________________	______________________________	____________________
Investigator/Delegate's Printed Name	Investigator/Delegate's Signature	Date

<u>Assistance Declaration</u>

Was the participant assisted during the consent process? ❑ Yes ❑ No

❑ The consent form was read to the participant/substitute decision-maker, and the person signing below attests that the study was accurately explained to, and apparently understood by, and consent was freely given by the participant/substitute decision-maker.

❑ The person signing below acted as a translator for the participant/substitute decision-maker during the consent process. He/she attests that they have accurately translated the information for the participant/substitute decision-maker, and believe that the participant/substitute decision-maker has understood the information translated.

Name of Person Assisting (Print)	Signature	Date
